# Glycometabolic Reprogramming of Microglia in Neurodegenerative Diseases: Insights from Neuroinflammation

**DOI:** 10.14336/AD.2023.0807

**Published:** 2024-05-07

**Authors:** Qi Huang, Yanfu Wang, Shanshan Chen, Fengxia Liang

**Affiliations:** ^1^Department of Rehabilitation, The Central Hospital of Wuhan, Tongji Medical College of Huazhong University of Science and Technology, Wuhan, China.; ^2^Key Laboratory for Molecular Diagnosis of Hubei Province, The Central Hospital of Wuhan, Tongji Medical College, Huazhong University of Science and Technology, Wuhan, China.; ^3^Department of Acupuncture and Moxibustion, Hubei University of Chinese Medicine, Wuhan, China

**Keywords:** microglia, metabolism, glycolysis, neuroinflammation, neurodegeneration

## Abstract

Neurodegenerative diseases (ND) are conditions defined by progressive deterioration of the structure and function of the nervous system. Some major examples include Alzheimer's disease (AD), Parkinson's disease (PD), and Amyotrophic lateral sclerosis (ALS). These diseases lead to various dysfunctions, like impaired cognition, memory, and movement. Chronic neuroinflammation may underlie numerous neurodegenerative disorders. Microglia, an important immunocell in the brain, plays a vital role in defending against neuroinflammation. When exposed to different stimuli, microglia are activated and assume different phenotypes, participating in immune regulation of the nervous system and maintaining tissue homeostasis. The immunological activity of activated microglia is affected by glucose metabolic alterations. However, in the context of chronic neuroinflammation, specific alterations of microglial glucose metabolism and their mechanisms of action remain unclear. Thus, in this paper, we review the glycometabolic reprogramming of microglia in ND. The key molecular targets and main metabolic pathways are the focus of this research. Additionally, this study explores the mechanisms underlying microglial glucose metabolism reprogramming in ND and offers an analysis of the most recent therapeutic advancements. The ultimate aim is to provide insights into the development of potential treatments for ND.

Neurodegenerative diseases (ND) are chronic, progressive conditions that cause the degeneration and degradation of neurons in the central and peripheral nervous systems. Notable examples include Alzheimer's disease (AD), Parkinson's disease (PD), and amyotrophic lateral sclerosis (ALS), which are often associated with various clinical symptoms, including cognitive impairments, memory deficits, and motor function disturbances. Neuroinflammation is believed to significantly impact the development and progression of these diseases [1, 2]. Initially, neuroinflammation facilitates tissue repair and assists in removing cellular debris, exerting a protective effect on tissue[3]. However, persistent or protracted neuroinflammation impedes regeneration and is a central pathogenic process in various neurological diseases. [4]. Extended inflammation leads to the activation of endothelial cells and undermines the integrity of the blood-brain barrier (BBB), consequently impairing the functionality of glial cells and neurons. Inflammatory mediators, including inflammatory cytokines, microRNAs, and extracellular vesicle-associated inflammatory components, disrupt neural repair pathways by negatively affecting the process of neurogenesis [5-7]. Despite extensive research on neuroinflammation's role in ND, the precise mechanisms involved in these disorders still require further elucidation.

Microglia, central nervous system (CNS) immune cells, have distinctive branching architectures that allow them to detect brain microenvironment changes [8, 9]. In ND, microglia become over-activated and release significant amounts of inflammatory mediators, generating an inflammatory microenvironment [10, 11]. After activation, microglia shift phenotype and continue to assess and remove brain tissue metabolites and debris [9]. They actively participate in synaptic remodeling, neuronal damage, and repair. These biological processes demand high energy within the brain [12]. The phenomenon where cells exhibit different energy metabolic profiles based on the energy and biomolecule requirements dictated by the microenvironment is known as metabolic reprogramming. Emerging research suggests that microglia metabolic reprogramming is critical to immune metabolism and neurodegeneration, notably involving glucose, amino acid, and fatty acid metabolism [13, 14].

Just like in other cells, glucose is the microglia's primary energy source [15]. Microglia activity is closely linked to glucose metabolism. Resting microglia produce adenosine triphosphate (ATP) for energy by oxidative phosphorylation (OXPHOS) [16]. However, microglia in a pro-inflammatory state exhibit a metabolic transition towards glycolysis, which is characterized by lower efficiency in energy production[16]. Conversely, anti-inflammatory microglia predominantly rely on OXPHOS. These discoveries provide significant understanding of the underlying mechanisms involved in microglia activation. Glycometabolic reprogramming in microglial cells may reduce neuroinflammation and prevent ND development [14]. However, the specific changes and metabolic pathways of microglial glucose metabolism in ND, and the mechanism of its action on neuroinflammation, are not yet fully understood.

Previous reviews have summarized the relationship between microglia metabolism and neuroinflammatory diseases. For instance, Lauro and Yang conducted reviews in 2020 and 2021 discussing the interplay between microglia metabolism and neuroinflammatory diseases [14, 17]. Similarly, Aldana and Paolicelli provided a comprehensive review in 2019 on the function and role of microglia metabolic disorders in neurological disorders [18, 19]. However, these reviews did not comprehensively address the topic of microglial glucose metabolism, including the crucial molecules and signaling mechanisms involved. This review aims to thoroughly investigate the characteristics of glucose metabolism during microglia activation in ND, encompassing the molecules and metabolic pathways implicated. Furthermore, the underlying mechanisms of microglial glucose metabolism in ND will be examined. Finally, we will summarize therapeutic advancements targeting microglial glycometabolic reprogramming in ND.

## Microglia and Neuroinflammation

Microglia, which comprise 5% to 12% of the brain in different locations, populate the brain parenchyma as the embryo develops from basic yolk sac myeloid progenitor cells [20, 21]. Their morphology is highly adaptable. In normal brain tissue, microglia exhibit a highly branched structure, commonly called "resting microglia" [22]. These cells establish direct contact with neuronal synapses, allowing them to precisely detect neuronal activation. In the presence of inflammation or injury in the brain, microglia undergo rapid activation [23]. Their protrusions disappear and become amoebic, allowing them to phagocytose [23]. Activated microglia migrate towards the site of injury, where they phagocytose cellular debris, damaged neurons, and pathogens [23].

Traditionally, microglia have been classified into two phenotypes upon activation: pro-inflammatory (M1) and anti-inflammatory (M2) [24, 25]. Pro-inflammatory microglia promote inflammation and neurotoxicity, while anti-inflammatory microglia limit inflammation and facilitate tissue healing [26]. However, this classification is now considered oversimplified as it fails to capture the intricate nature of alterations within the inflammatory response. During the initial phases of central inflammation, microglia undergo activation and polarization towards a pro-inflammatory phenotype, thereby participating in the phagocytosis and subsequent elimination of necrotic neurons and cellular debris. This microglia phenotype reduces the release of toxic compounds like inflammatory cytokines and chemokines, thereby protecting the brain [25]. However, if this phenotype becomes dominant, there is a notable decrease in their phagocytic capabilities, accompanied by an increase in the secretion of inflammatory cytokines and other neurotoxic mediators. This leads to the destruction of BBB, neuronal injury, and dysfunction [27-29]. On the contrary, microglia stimulated by anti-inflammatory cytokines such as interleukin (IL)-4, IL-10, and transforming growth factor (TGF)-β, exhibit a propensity towards promoting neurogenesis or enhancing phagocytosis [29-31]. An illustration of this phenomenon can be observed in the neurotrophic factor insulin-like growth factor (IGF)-1, which has been detected in anti-inflammatory microglia and potentially plays a role in the process of neurogenesis [32]. However, research conducted on models of ischemic stroke and cerebral hemorrhage has demonstrated the coexistence of M1 and M2 phenotypes of microglia at the site of injury, with comparable timelines of activation and polarization [33, 34]. This finding implies a potential antagonistic relationship between these two phenotypes, wherein they collaborate to maintain the homeostasis of the body's inflammatory response. Furthermore, microglia have the ability to transition between phenotypes in response to changes in the CNS environment [32, 35], thereby exerting neuroprotective effects. In the context of neurodegeneration, microglia exhibit a range of intermediate phenotypes, highlighting the inadequacy of the M1/M2 paradigm in accurately describing microglia activation [29].

Microglia emit pro- or anti-inflammatory cytokines under complex conditions, depending on disease stage, inflammation, and regulatory interaction. Commonly involved factors in microglia activation include tumor necrosis factor-alpha (TNF-α), IL-6, IL-4, IL-10, and interferon-gamma (IFN-γ), among others [25, 28, 29]. Molecular analysis of these molecules provides valuable insights into microglia activation and inflammatory responses. However, the specific methods for inducing a desirable state of microglia activation remain unidentified. Research is needed to understand microglia activation and establish settings that promote a healthy phenotype.

Energy metabolism is essential for the activation and functional integrity of microglia. In an activated state, microglia experience an increased demand for energy, leading to enhanced glucose uptake and metabolism to meet their energy requirements. Consequently, microglial glucose metabolism changes in response to the environment stimulated by chronic inflammation.

The functional activity of microglia relies on various membrane proteins, including immune receptors and pattern recognition receptors (PRRs) like toll-like receptor (TLR), triggering receptors expressed on myeloid cells 2 (TREM2), and fractalkine receptor (CX3CR1) [36, 37].

These receptors are responsible for recognizing neurodegeneration-associated molecular patterns (NAMPs), damage-associated molecular patterns (DAMPs), and pathogen-associated molecular patterns (PAMPs) [36, 38]. Among these receptors, TREM2 is critical in the signaling pathway primarily influenced by NAMPs. The ligands and pathways mediated by TREM2 are crucial for microglial response to signals associated with ND [39]. DAMPs, rather than PAMPs, activate microglia within the brain parenchyma due to the presence of the BBB [8]. Consequently, changes in glucose metabolism induced by PAMP- and DAMP-activated microglia also exhibit divergent characteristics.

## Glucose metabolism of microglia in neuroinflammation

The intake and usage of glucose, the brain's principal energy source, are strictly regulated. Neurons and astrocytes consume most glucose in the brain under normal conditions [40]. However, in specific pathological conditions like neuroinflammation and neurodegeneration, microglia undergo activation and reprogramming of CNS glucose metabolism. This reprogrammed metabolic pathway increases glucose absorption, anaerobic glycolysis, and oxidative pentose phosphate pathway (PPP) activation while maintaining mitochondrial function [41]. These changes enable microglia to bolster their defense capability, ensuring optimal CNS signaling function.

### Glucose metabolic pathways in microglia

The energy requirements of microglia primarily stem from the catabolic processes of various nutrients, including glucose, lipid, and protein. The critical process for energy production is OXPHOS [42]. Glucose follows the glycolytic pathway, broken down into pyruvate by pivotal enzymes like hexokinase (HK), phosphohexose isomerase, and phosphofructokinase (PFK). Under normal oxygen conditions (normoxia), pyruvate undergoes decarboxylation across the mitochondrial membrane, which yields acetyl-coenzyme A (Acetyl-CoA). This intermediate then enters the tricarboxylic acid (TCA) cycle, generating ATP, a vital energy molecule [42]. Conversely, under anaerobic conditions where oxygen is lacking, pyruvate undergoes reduction to lactate through the action of lactate dehydrogenase (LDH) in the cytosol, resulting in a restricted energy yield [17]. The PPP directly involves glucose in oxidative dehydrogenation and decarboxylation processes, producing reduced nicotinamide adenine dinucleotide phosphate (NADPH) and mitochondrial reactive oxygen species (ROS) [43]. These byproducts contribute to fatty acid and cholesterol biosynthesis, making this another important function of glucose beyond energy provision.

**Figure 1.**
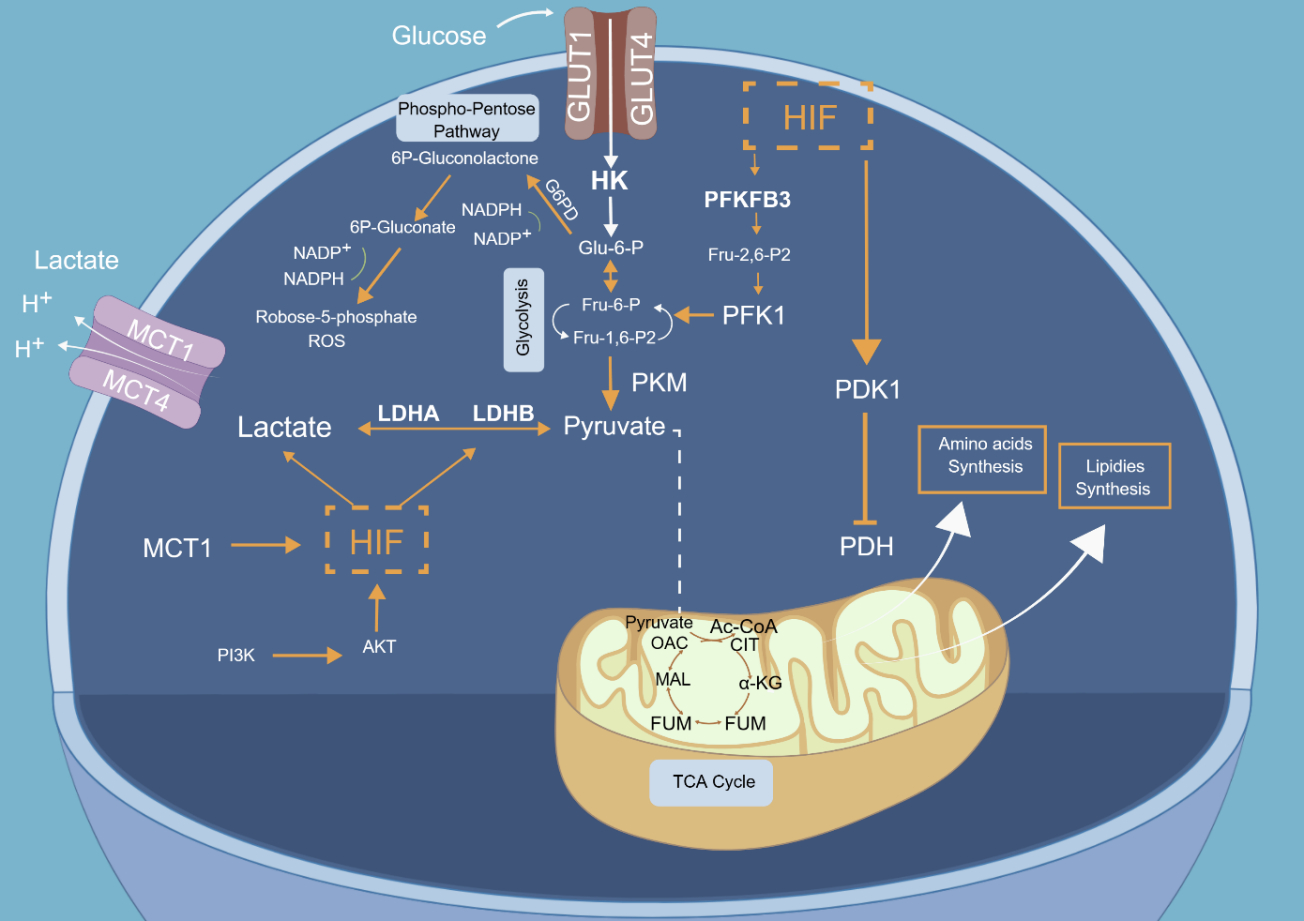
**Glucose metabolic pathways and molecular determinants in microglia**. Under physiological conditions, microglia primarily use the OXPHOS pathway to produce ATP through glucose metabolism. Glucose is transported into the cell by GLUTs and converted into glucose-6-phosphate by HK. Subsequently, PFK and PKM2 enzymes help generate pyruvate, which is converted to Acetyl-CoA by PDH. Acetyl CoA then enters the TCA cycle to produce ATP. In inflammatory conditions, microglia switch from OXPHOS to glycolysis, and LDH converts pyruvate to lactate, producing ATP. Lactate is transported extracellularly by MCT, and HIF-1α regulates these metabolic changes. Pathological conditions contribute to an increase in the pentose phosphate pathway in microglia, leading to NADPH and mitochondrial ROS generation. The figure was created using www.figdraw.com.

Aerobic glycolysis assumes significance as a pivotal alternative route when cells confront obstacles in energy supply. This process entails augmenting glucose absorption and glycolytic function in the presence of oxygen, resulting in increased pyruvate generation. Surplus pyruvate is subsequently metabolized into lactate within the cytosol to meet energy requirements, a phenomenon commonly known as the "Warburg effect" (Fig. 1) [44].

### Molecular determinants in glucose metabolism in microglia

A lot of data suggests metabolic reprogramming in microglia after activation during neurodegenerative disorders, but molecular pathways remain unclear. The following section provides an overview of key molecules that regulate alterations in microglial glucose metabolism during inflammatory conditions and describes their respective modes of action.

#### Glucose transporters (GLUTs)

Brain glucose uptake depends on microglia-mediated inflammatory responses [45]. In a "resting" state or when differentiated into an anti-inflammatory phenotype, microglia rely on OXPHOS for energy production. They switch from OXPHOS to glycolysis and become pro-inflammatory phenotypes when exposed to inflammatory stimuli [44, 46]. Pro-inflammatory microglia primarily utilize glycolysis as their energy source, necessitating increased glucose uptake. It relies on various GLUTs for glucose to enter the cell. Microglia expresses various GLUTs, including GLUT1, GLUT3, GLUT4, and GLUT5. Notably, inflammatory stimuli elicit a particularly high expression of GLUT1 [47]. Upon stimulation with lipopolysaccharide (LPS), pro-inflammatory BV-2 microglial cells significantly increase GLUT1 expression, resulting in elevated glycolytic activity and the release of lactate, a major byproduct of glycolysis [48]. Inhibiting GLUT1 expression using STF31, a specific inhibitor, decreases glucose uptake by microglia and reduces the expression of pro-inflammatory cytokines like IL-1β, TNFα, and IL-6 [47]. This suggests a link between glucose absorption and microglia inflammatory expression. GLUT1 is vital in reprogramming glucose metabolism, facilitating increased glucose transport and elevated glycolytic flux.

#### Type II hexokinase (HK2)

The HK enzyme catalyzes the initial step of glucose metabolism, converting glucose into glucose-6-phosphate (G6P). Mammalian organisms exhibit the expression of various isoforms of HK, specifically HK1, HK2, HK3, and HK4 [49]. Among these isoforms, HK2 assumes a crucial dual function in the regulation of glucose metabolism in microglial cells. First, it directly controls the flow of glycolysis and energy generation, impacting the microglial physiological function. Second, it interacts with the outer mitochondrial membrane to modulate the mitochondrial activity and immune response of microglia, contributing to the development of pathological conditions[50]. HK2 expression in microglia is necessary for mitochondrial glycolysis and glycolysis-derived oxidative respiration. It facilitates the maintenance of the mitochondrial membrane electrochemical potential difference (Δψ), preventing excessive generation of ROS and ROS-induced immune responses[51]. Deficiency of HK2 substantially promotes microglia activation, phagocytosis, and inflammatory responses [49, 51]. Thus, understanding HK2's role in microglia homeostasis and disease development will illuminate CNS microglia metabolism.

#### Fructose-6-phosphate 1-kinase (PFK1)

PFK is an enzyme that catalyzes the third step of glycolysis, regulating glycolytic rate by transforming fructose 6-phosphate (F6P) into fructose 1,6-bisphosphate (F1,6BP). It comprises multiple isoforms, with PFK1 being the primary regulatory enzyme of glycolysis. One specific isoform, namely 6-phosphofructo-2-kinase/fructose-2,6-bisphosphatase isoform 3 (PFKFB3), functions as a fructose-6-phosphate 2-kinase (PFK2) isoform and is regulated by the activator fructose-2,6-bisphosphate. PFKFB3 controls the glycolytic flow, as determined by measurements of intracellular PFK1 concentration [52].

LPS-induced early inflammatory changes increase microglia PFK1 activity and lactate output. This, in turn, causes a reduction in the secretion of IL-1β and S100B, a protein synthesized and secreted by astrocytes [53]. In microglia polarized by IFN-γ, there are characteristic elevations in TNFα and nitric oxide synthase (NOS) 2, accompanied by upregulated expression of PFKFB3 and enhanced glycolysis [54]. PFKFB3 expression inhibition in microglia stimulated by LPS + amyloid-beta (Aβ) results in the restoration of oxidative metabolism and reduction in inflammasome activation, leading to improved phagocytosis recovery [16]. Overall, PFK1 and PFKFB3 exert significant roles in microglial glycolysis regulation.

#### Monocarboxylate transporter (MCT)

The MCT is a crucial transmembrane protein for transporting monocarboxylates including pyruvate, lactate, and ketone bodies [55, 56]. Immunohistochemical studies have demonstrated abundant expression of MCT isoforms 1, 2, and 4 in the CNS [55, 56]. MCT is vital in neuroenergetics and regulation of glucose homeostasis in the brain. Although the investigation of MCT expression in microglia is not exhaustive, recent reports confirm its presence in these immune cells. During ischemia, activated microglia exhibit more MCT1 and MCT2 expression, providing an energy source for their activation. Notably, there is also a discernible augmentation in the expression of MCT1 and MCT4, accompanied by substantial levels of PFKFB3, within activated microglia [57]. Inhibiting MCT1 reduces microglial glycolysis and mitigates the effects of LPS-induced IL-1β, IL-6, and iNOS [58]. This mechanism is believed to be associated with the downregulation of HIF-1α expression, a crucial factor in glycolysis[58]. Hence, MCT1 and PFKFB3 may promote glycolysis and enhance microglia polarization induced by LPS.

In cancer, MCT proteins are vital in the glycogen metabolic pathway due to their transport abilities, making them potential targets for tumor therapy [59]. Some breakthroughs have already been achieved in this field [59]. However, MCT's role in brain illnesses needs further study. Consistent with existing studies, small molecule drugs and MCT1 inhibitors hold promise for treating ND [60].

#### Pyruvate kinase M2(PKM2) and Pyruvate dehydrogenase (PDH)

PKM2, the final rate-limiting enzyme in the glycolytic pathway, produces pyruvate. It converts phosphoenol-pyruvate to pyruvate. PDH is essential for the Acetyl-CoA production at equilibrium. ATP synthesis during glycolysis or OXPHOS depends on both enzymes. Drugs inhibiting PKM2 or PDH prevent microglia from producing ATP [61].

Interestingly, PKM2 levels are specifically elevated in microglia adjacent to Aβ plaques in human AD brain tissue samples and animal models [62]. Large-scale proteome investigations reveal that AD samples with higher PKM2 levels activate microglia and astrocytes [63]. This implies that the enzymes participating in the glycolytic pathway play a role in the inflammatory response triggered by the activation of microglia. PKM2, a crucial metabolic adaptor in pro-inflammatory macrophages [64], is also expected to modulate the activation status of microglia by influencing their metabolic alterations and thereby regulating neuroinflammation. A study substantiated this notion by demonstrating the interaction between PKM2 and activating transcription factor-2, a transcriptional activator that fosters inflammation, thereby establishing a connection between aerobic glycolysis, pyroptosis in microglia, and the promotion of neuroinflammation [65].

Similarly, PDH produces a comparable effect in ND. In a mouse model of PD, the melatonin receptor 1 (MT1) positively regulates the expression of pyruvate dehydrogenase α1 (PDHα1) in pro-inflammatory microglia in the substantia nigra. This reversal of LPS-induced neuroinflammation counters excessive aerobic glycolysis and impaired OXPHOS [66]. PKM2 and PDHα1 expression levels alter microglial activation and metabolic state. Targeted therapies to modify microglia enzyme expression and glucose metabolism may cure neuroinflammation illnesses.

#### Lactate dehydrogenase (LDH)

In most cases, LDH occurs as a homo- or heterotetrameric complex that consists of two subunits, LDHA and LDHB, in different combinations [67]. This enzyme is vital in the glycolytic pathway by facilitating the reduction of pyruvate to lactate, while also converting NADH to NAD+ in order to sustain glycolytic flux [68]. Recent research suggests that microglia possess the necessary components, including LDH, required for efficient lactate metabolism [69]. However, it is important to note that the functions of the two subunits of LDH may not always be identical. In the presence of oxygen, LDHB exhibits the ability to catalyze the conversion of lactate to pyruvate, enabling its entry into the TCA cycle and subsequently enhancing ATP production[70]. Conversely, under anaerobic conditions, LDHA demonstrates a heightened affinity for pyruvate, leading to its conversion into lactate and resulting in the oxidation of NAD+ to NADH [67, 70]. In microglia, LDHB is significantly expressed [71, 72], particularly in the adult hippocampus, where it is mostly exclusive to this cell type compared to others [73].

These results indicate that the glycolytic pathway may serve as the primary metabolic route in activated microglia. In BV-2 cells treated with LPS, glycolysis is vital for ATP generation, resulting in the release of lactate[74, 75]. This suggests that LDH potentially governs the production of lactate in microglia, particularly during proinflammatory reactions, to ensure optimal functionality in response to environmental stimuli. Consequently, LDH plays a significant role in regulating microglial glucose metabolism, which is crucial for maintaining proper nervous system function.

### Signaling pathway for glucose metabolism in microglia

**Figure 2.**
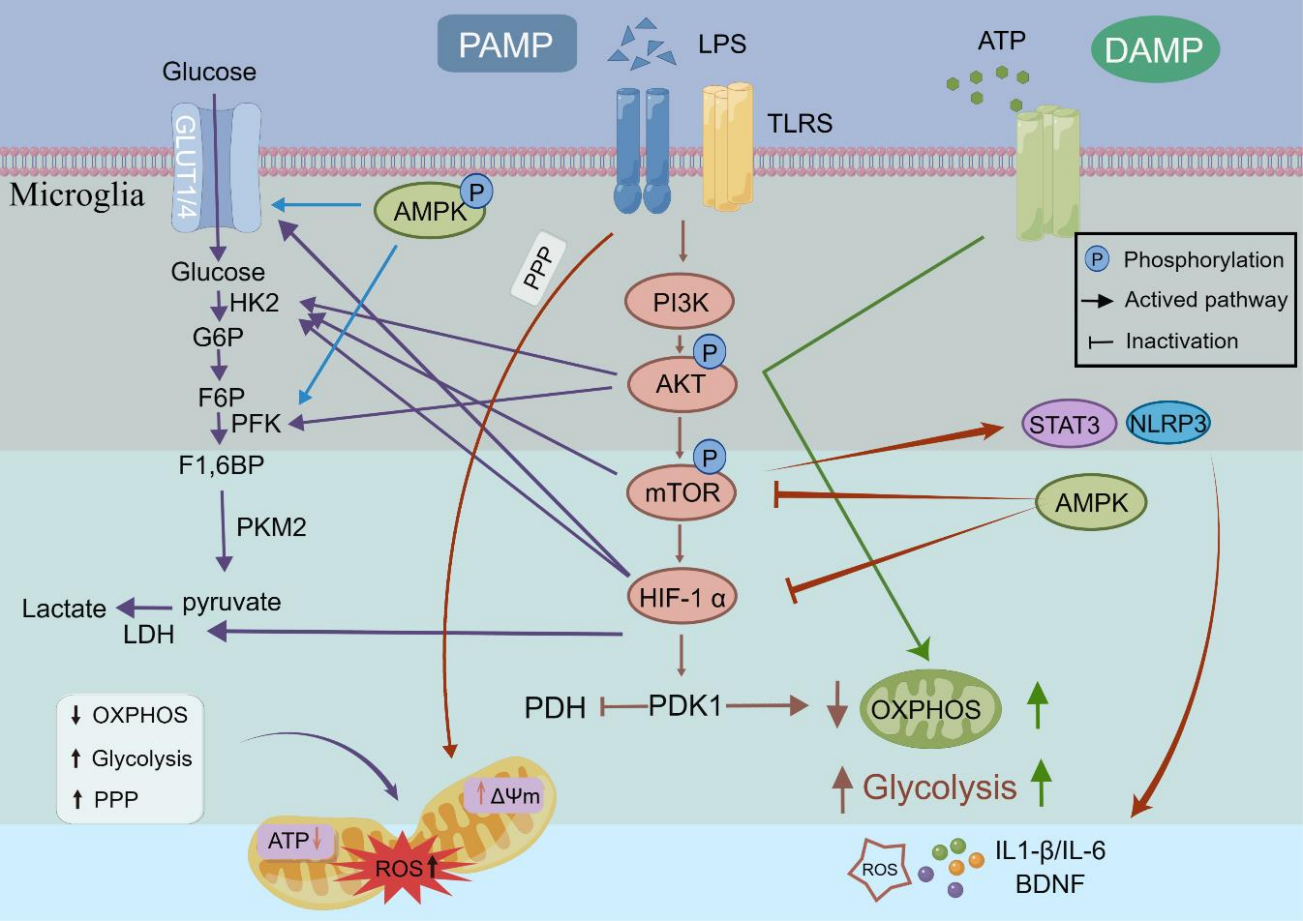
**Molecular mechanisms for glycometabolic reprogramming in microglia**. Upon LPS/ATP exposure, microglia activate TLRs, which, in turn, activate AKT-mTOR signaling pathway, upregulating HIF-1α, GLUT1, and glycolysis-related enzymes (HK2, PDK, LDH). Consequently, reduced OXPHOS leads to ROS production, mitochondrial fission, and inflammatory factor secretion. Additionally, the AKT-mTOR signaling pathway directly activates NLRP3 or STAT3, promoting inflammatory transcription and further glycolysis. AMPK phosphorylation increases glucose utilization, whereas inhibition of AMPK activity induces mTOR/HIF-1α and stimulates glycolysis. LPS exposure further activates the pentose phosphate pathway, exacerbating mitochondrial dysfunction and impairing ATP production in microglia. The figure was created using www.figdraw.com and some components were assembled in Adobe Illustrator.

Microglia can modulate neuroinflammatory responses and the inflammatory process's progression and resolution through glucose metabolic pathways. The modulation of microglial inflammatory responses to glucose metabolism involves multiple signaling pathways. In the subsequent sections, we will explore some of the crucial signaling pathways and mechanisms that govern the rewiring of microglial glucose metabolism in the inflammatory microenvironment (Fig. 2).

#### Mammalian target of rapamycin(mTOR)

The mTOR protein consists of two distinct complexes: rapamycin complex 1 (mTORC1) and rapamycin complex 2 (mTORC2) [76]. These complexes play a role in regulating the expression or activity of GLUTs, glycolytic enzymes, transcription factors, and glycolytic effectors [77]. Activation of mTOR is essential for microglia to effectively utilize glucose as an energy source and to respond to inflammatory stimuli[78]. Studies have shown that certain PAMPs, such as LPS, and DAMPs, like ATP, can activate mTOR [8]. Furthermore, mTOR directly influences the expression of HK1 and NOD-like receptor protein 3 (NLRP3), resulting in an augmented secretion of the inflammatory factors IL-18 and IL-1β[79]. Inhibiting mTOR expression suppresses NLRP3 activation in microglia stimulated by LPS, highlighting the essential role of mTOR-induced glycolysis in the activation of inflammatory processes [79]. Additionally, inhibiting mTOR activity reduces glycolysis and intracellular ROS production, as well as decreases the expression of proinflammatory cytokines and brain-derived neurotrophic factor (BDNF) stimulated by LPS and ATP in microglia[8]. Thus, mTOR is a key target in central inflammation-related illnesses like ageing and neurodegeneration, and understanding its activities and complexes is essential for developing more effective therapeutic options.

#### Hypoxia-inducible factor-1 alpha (HIF1-α)

Hypoxia-inducible factor (HIF) is the primary intracellular oxygen sensor, comprising two subunits: inducible HIF-α and structural HIF-β. HIF activation during hypoxia is essential for optimal intracellular ATP synthesis and is associated with ROS generation, either directly or indirectly. HIF activation inhibits the function of the mitochondrial TCA cycle, resulting in a reduction of ROS formation [80]. Alternatively, ROS production can increase by upregulating the target gene NADPH oxidase (NOX), an enzyme responsible for generating superoxide, within the HIF pathway [80]. HIF-1α, acting downstream of mTOR, plays a pivotal role in regulating metabolic reprogramming and functional phenotype of microglia, whether pro- or anti-inflammatory [81]. Under normoxic conditions, the abnormal activation of HIF-1α results in a metabolic shift towards aerobic glycolysis. The activation of microglia into a pro-inflammatory state triggers the activation of mTOR and the upregulation of HIF-1 expression [82]. Consequently, this leads to heightened activity of GLUT1 and glycolysis-associated enzymes such as HK2, LDH and phosphoglycerate kinase 1 (PGK1), culminating in a reduction in microglial OXPHOS and an elevation in ROS production [82]. The accumulation of ROS further stabilizes HIF-1α activity, thereby promoting the glycometabolic reprogramming of microglia [82]. Prolonged and excessive activation of HIF-1α results in the arrest of the microglial cell cycle, impaired proliferation, and aggregation around Aβ plaques, ultimately exacerbating neurological damage [83]. These findings suggest that HIF-1α governs microglial polarization status through the regulation of glycolysis. Notably, important glycolysis-related genes, such as GLUT1, HK2, and PFKFB3, are direct targets of HIF-1α, underscoring the close relationship between HIF-1α and microglial glycolysis [84].

#### PI3K-AKT-mTOR

The phosphatidylinositol 3-kinase/serine threonine kinase (PI3K-AKT) signaling pathway plays a significant role in regulating microglial glycolysis, impacting crucial processes including glucose metabolism, apoptosis, and transcription. Stimulation of microglia through LPS activation initiates the activation of the PI3K-AKT pathway, resulting in heightened glycolysis and the promotion of inflammation associated with ND [85]. The activation of AKT, a regulatory protein upstream of the mTOR, leads to the phosphorylation of mTOR and subsequent increased expression of HIF-1α in microglia [86]. The elevated HIF-1α levels enhance the transcription of pyruvate dehydrogenase kinase 1 (PDK1), which stimulates glucose uptake and glycolysis while inhibiting OXPHOS and ROS production. This exacerbates the detrimental effects of Aβ toxic injury [87]. Inhibiting the AKT-mTOR signaling pathway reduces the phosphorylation of the activator of transcription 3 (STAT3) and mitigates microglia-mediated neuro-inflammation [88].

#### Serine/threonine kinase AMP-activated protein kinase (AMPK)

AMPK is a well-known metabolic molecule that is crucial in regulating OXPHOS. Activation of AMPK governs four major categories of mammalian metabolism, namely glucose metabolism, lipid metabolism, protein metabolism, and autophagy and mitochondrial homeostasis [89]. This comprehensive control extends to nearly all physiological and metabolic functions in living organisms. Notably, AMPK phosphorylation facilitates the translocation of GLUT1 and GLUT4 to the plasma membrane, thereby enhancing cellular glucose utilization [89]. Additionally, AMPK exerts regulatory control over glycolysis by the phosphorylation of PFKFB3, thus, influencing the activity of the rate-limiting enzyme PFK1[89].

Pertinent findings from investigations conducted in adult rodents indicate that macrophages, when stimulated by anti-inflammatory factors such as IL-4, STAT3, and IL-10, exhibit a preference for activating OXPHOS, resulting in the expression of the AMPK and subsequent development of an immunosuppressive phenotype[90]. Conversely, pro-inflammatory mediators such as LPS exhibit a preference for stimulating glycolysis and inhibiting the upregulation of AMPK in macrophages[90, 91]. The activation of AMPK leads to the phosphorylation of key proteins in various signaling pathways, including mTORC1, resulting in an increase in catabolism and a decrease in anabolism [92, 93]. AMPK inhibition in microglia leads to an increase in mTOR-HIF-1α signaling, glycolysis, and inflammatory factor mRNA expression [94]. This suggests that the metabolic function of AMPK in microglia aligns with that in macrophages. Therefore, inhibiting the expression of AMPK may yield positive therapeutic effects in reversing microglia glycolysis. Understanding AMPK's function and downstream targets is crucial for regulating microglia metabolism in inflammatory states.

#### Mitochondrial dysfunction

Mitochondria, being the sole entities with genetic influence beyond the nucleus, possess an independent and unique circular genome. Due to the lack of histone safeguarding, mitochondrial DNA is exceptionally susceptible to diverse forms of harm, particularly from ROS produced in close proximity.

Upon LPS exposure, primary microglia demonstrate an exaggerated process of mitochondrial fission, resulting in the fragmentation of mitochondria[74]. Following activation of TLR4, microglia undergo metabolic reprogramming from OXPHOS to glycolysis. However, the use of Mdivi-1, an inhibitor of mitochondrial fission, can reverse this metabolic shift [74]. Consequently, this reversal affects mitochondrial membrane potential, ROS generation, TCA cycle intermediate accumulation, and inflammatory factor expression [74]. These findings emphasize the crucial role of mitochondrial morphology in maintaining their functionality and highlight the significance of mitochondria in cellular processes.

Moreover, microglia that have been subjected to LPS and IFN-γ treatment display a metabolic alteration favoring the PPP, which is evidenced by heightened activity of glucose-6-phosphate dehydrogenase (G6PD), a pivotal enzyme in the PPP [41, 95]. This metabolic shift results in augmented synthesis of NADPH, mitochondrial ROS, and nitric oxide (NO) levels [41, 95]. The prolonged accumulation of NO can competitively bind to the oxygen-binding site of Complex IV in the mitochondrial respiratory chain, leading to abnormalities in mitochondrial membrane potential and contributing to the progression of ND [96]. Thus, mitochondrial dysfunction-mediated glucose metabolic reprogramming is connected to pro-inflammatory microglia activation, suggesting the potential of targeting this process for therapeutic.

## Role of microglial glycometabolic reprogramming in ND

**Figure 3.**
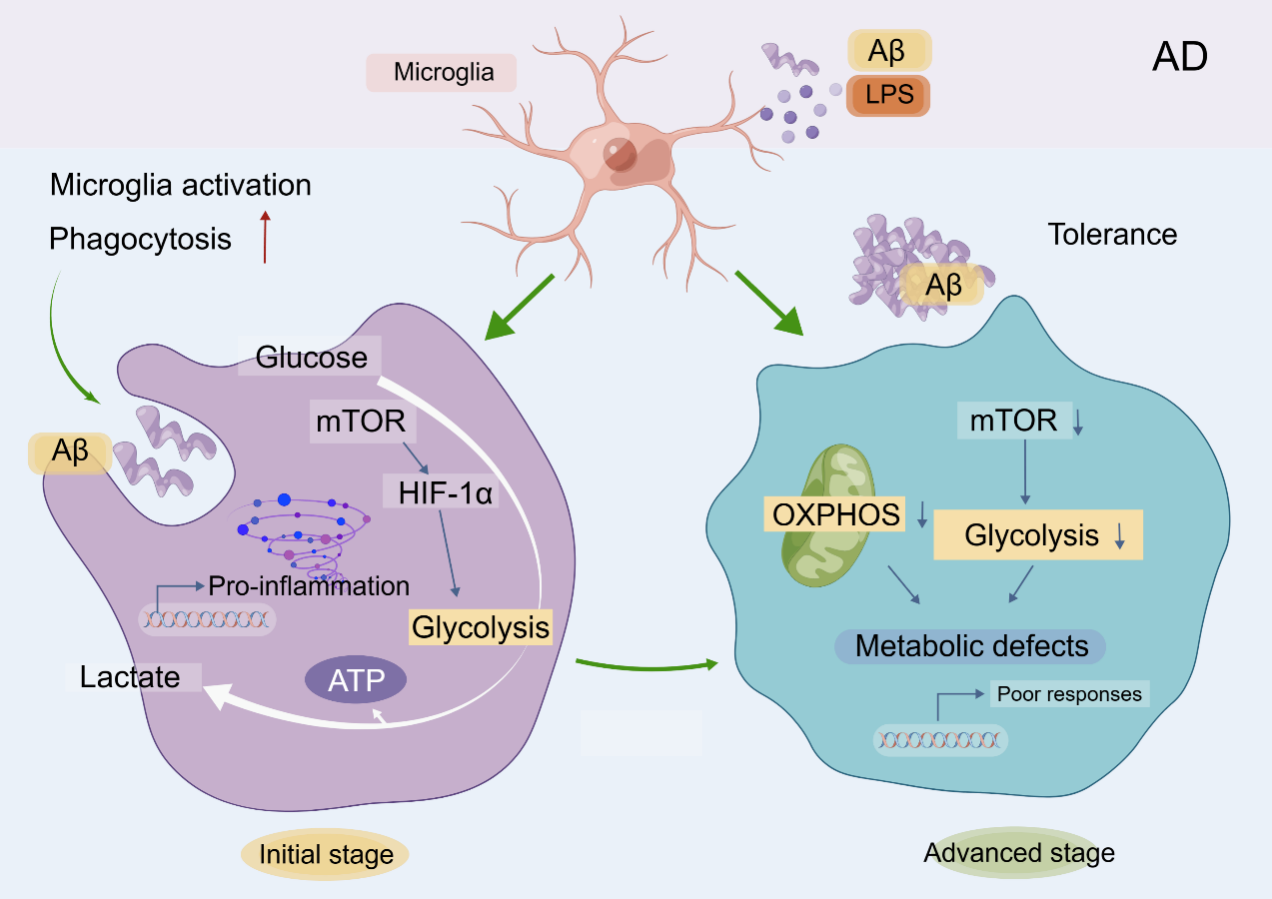
**Glycometabolic reprogramming of microglia in different stages of AD**. In the initial stages of AD, activated microglia initially switched from OXPHOS to glycolytic metabolism, with increased glucose uptake, mTOR pathway activation, elevated HIF-1α expression, and increased pro-inflammatory cytokine production. In contrast, in advanced AD stages, microglia become tolerant immune cells, with deactivated mTOR signaling, impaired glycolysis and OXPHOS, diminished phagocytosis, and reduced inflammatory response to Aβ. The figure was created using www.figdraw.com.

*AD:* AD is a prevalent neurodegenerative disorder characterized by the accumulation of extracellular plaques containing the Aβ protein and the formation of intracellular tangles composed of heavily phosphorylated tau proteins [97]. The cognitive impairments observed in AD are associated with irregularities in cerebral glucose utilization, glycolysis, and oxygenation metabolism [98, 99]. Additionally, neuroinflammation manifests in the early stages of AD progression and plays a significant role in the pathogenesis of the disease[100]. This combination of factors leads to increased microglial glycolysis, impaired ATP production, elevated release of ROS, mitochondrial fission, fragmentation, and extracellular release [101]. In AD, microglia sense Aβ peptides and clear them while monitoring the intracerebral environment. The mTOR signaling pathway activates, HIF-1α expression rises, microglial metabolism flips from OXPHOS to glycolysis, proinflammatory cytokines increase, microglial phagocytosis activity increases, and microglia move to the lesion site to phagocytose Aβ [82, 100]. However, as the disease progresses, microglia become immune-tolerant, the signaling pathway becomes inactivated, glucose metabolism becomes defective, the inflammatory response is reduced, and microglia lose their phagocytic activity while becoming unresponsive to Aβ [82]. Positron emission tomography (PET) studies have revealed increased reliance on aerobic glycolysis in certain AD brain regions associated with Aβ deposition [102, 103] (Fig. 3).

Furthermore, Tau proteins bind to mitochondria, causing mitochondrial toxicity and metabolic dysfunction, preventing OXPHOS and ATP generation [104]. A protein network study suggests that glycolysis may improve AD pathophysiology [63]. These findings highlight the significance of impaired microglial OXPHOS and glycometabolic reprogramming as potential targets for AD treatment strategies.

*PD:* Lewy bodies and neurites containing α-synuclein describe PD as the second most common ND [105]. Additionally, damage to dopaminergic neurons in the substantia nigra and other brain regions contributes to PD development [105]. PET studies have demonstrated prevalent activation of microglia in intracerebral regions during the initial phases of PD [106]. This activation is triggered by the presence of extracellular α-synuclein, leading to subsequent phagocytosis[106, 107]. The activation of microglia also induces NOX activation and the generation of ROS [108, 109]. Notably, both human and animal models of PD have exhibited a significant upregulation of G6PD expression in microglia, an increase in the PPP, and an elevated metabolic production of NADPH [95]. These metabolic alterations excessively activate NOX2, leading to ROS generation, denaturation, and deactivation of dopamine (DA) neurons [95]. Furthermore, the reaction between NOX2 and NO produces peroxynitrite, which contributes to neuronal death[95]. On the other hand, activated microglia shift from OXPHOS to aerobic glycolysis via the AKT-mTOR-HIF-1α pathway. However, over time, activated microglia transition to a state tolerant of α-synuclein, exhibiting a significant defect in energy metabolism in the PD’s brain [44].

Clk1, an important component of the mitochondrial respiratory chain and antioxidant resistance, is vital in electron transport. Clk1-deficient microglia rely on the AMPK-mTOR-HIF-1α and ROS-HIF-1α signaling pathways, thereby increasing the inflammatory response mediated by aerobic glycolysis and contributing further to dopaminergic neuronal damage [110]. Thus, microglial glycometabolic reprogramming in PD and the stage at which it causes permanent dopaminergic neuronal death must be studied.

*ALS:* An ALS diagnosis is accompanied by symptoms of lower and upper motor neuron degeneration along the corticospinal pathway. This illness weakens and causes specific skeletal muscles' atrophy, eventually paralyzing them [111]. Research on adult family lineages has identified specific gene mutations, including SOD1, C9orf72, TDP43, and FUS74, which are associated with the heritability of ALS [112, 113]. Among these mutations, SOD1 is the most common form of inherited ALS. In human and animal models of ALS, pathologically affected brain and spinal cord regions exhibit neuroinflammation characterized by large aggregates of activated microglia [114, 115]. Mutant SOD1 microglia produce more NADPH-oxidized ROS [116]. Inhibition of the SOD1 mutation has been shown to preserve mitochondrial function in ALS, reducing mitochondrial ROS formation and enhancing ATP synthesis [116]. However, existing studies on ALS metabolism have predominantly focused on neurons and astrocytes [117, 118]. Future studies must clarify ALS-associated microglial cell glucose metabolism.

In summary, the inflammatory environment in ND leads to microglia activation and phenotypic alterations. This activation influences the glucose metabolism pathway in microglia, thereby affecting neuronal function and survival. Table 1 provides a compilation of microglia activation modes in glycometabolic reprogramming in ND. Understanding these activation modalities and alterations in glucose metabolism may help explain ND's pathogenesis.

**Table 1:** The roles of microglia in glycometabolic reprogramming of ND.

Metabolic pathway	Neurological disorders	Microglial phenotype	Associated factors	Ref.
**Glycolysis**	AD	Pro-inflammatory	TNF-α, iNOS, IL-1β	[62, 119]
**Increased aerobic glycolysis** **Inhibited OXPHOS**	AD	Pro-inflammatory	TNF-α, IL-1β	[82, 166]
**OXPHOS to Glycolysis**	AD	Anti- inflammatory Pro-inflammatory	IL-10; TNF-α, IL-6	[142]
**Anaerobic glycolysis to OXPHOS**	AD	Anti- inflammatory Pro-inflammatory	IL-4, IL-10, Aβ clearance TNF-α, IL-1β, IL-6	[163]
**Aerobic glycolysis to OXPHOS**	PD	Anti- inflammatory Pro-inflammatory	TNF-α, iNOS, IL-1β, COX-2	[66]
**Glycolysis**	AD	Phagocytosis phenotype	MHCII, CD68, CD11c, CD45	[159]
**Glycolysis**	ND	Anti- inflammatory	TNF-α, Nos2, IL-1β, IL-6	[120]
**Glycolysis to OXPHOS**	ND	Anti- inflammatory Pro-inflammatory	TNF-α, IL-1β, IL-6	[121]
**Pentose phosphate pathway**	AD	Anti-inflammatory Pro-inflammatory	TNF-α, IL-1β	[154]
**Mitochondrial dysfunction**	HD	Pro-inflammatory	iNOS	[167]
**OXPHOS to glycolysis**	AD	Pro-inflammatory	TNF-α	[168]
**Glycolysis**	Aging	Pro-inflammatory	IL-1β	[169]

## Mechanisms of microglial glycometabolic reprogramming in ND

### Microglia activation influences glucose metabolism

The microglia activation in ND is influenced by microenvironmental stimuli and metabolic changes. Microglial activation can be triggered by misfolded proteins, such as those found in AD or PD, which directly interact with microglial metabolism [101, 107]. The immune function of microglia relies on ATP for proper functioning, and in response to inflammatory stimuli, pro-inflammatory microglia transition from OXPHOS to aerobic glycolysis [46]. Glycolysis is less efficient at producing ATP, but it is fast at metabolizing glucose and supplying microglia with energy. The metabolic shift results in an increase in the release of proinflammatory products by microglia (e.g., TNF-α and IL-1β), further exacerbating CNS inflammation [62, 119-121]. Consequently, the diminished metabolic efficiency of pro-inflammatory microglia forms the basis for reduced microglial phagocytosis. Consistent with the research conducted by Baik et al, the alteration in microglial cell metabolism towards aerobic glycolysis and the heightened secretion of inflammatory cytokines in mouse models of AD were concomitant with an escalation in Aβ plaque accumulation[82]. This phenomenon signifies the impaired capacity of microglia to phagocytose pathological proteins like Aβ, leading to their accumulation in the brain and facilitating the progression of the disease [82]. However, subsequent to the reactivation of microglia through the utilization of INF-γ, microglia were able to restore their mobility and phagocytic capacity towards Aβ, while simultaneously undergoing glycolysis [82]. This restorative process effectively mitigated cognitive impairment in mice with AD. These findings indicate that the modulation of cellular glucose metabolism pathways can significantly impact the functional behavior of microglia, leading to alterations in both activation and "resting" states. Thus, the intimate association between microglial cell metabolism and their functional phenotype becomes apparent.

### Glycolysis regulates microglia inflammatory signaling

Inflammation-induced glycometabolic reprogramming in microglia involves many molecular mechanisms. The regulation of various inflammatory signals in microglia, and their pro- or anti-inflammatory properties, rely on glycolysis. In AD model mice, microglia exhibit activation and engage in aerobic glycolysis, which is contingent upon the activation of the mTOR-HIF1α pathway[82]. Within microglia, pro-inflammatory factors like TNF-α and IL-6 activate inflammatory signaling pathways including nuclear factor-kappa B (NF-κB), activator protein 1 (AP-1), and STAT3 [46, 122, 123]. These signaling pathways interconnect with mTOR, thereby assuming a regulatory role in the metabolic processes of microglial cells in ND. The activation of mTOR triggers the expression of HIF1α and is controlled by AKT and AMPK signaling pathways. This activation results in heightened activity of crucial glycolytic enzymes, including GLUT1, HK2, and PDK1, leading to the reprogramming of microglial cell glycolysis [82, 86, 94]. This sustained increase in glycolysis serves a dual role: firstly, it facilitates lactate production to supply energy and metabolic assistance to neurons[124, 125]; secondly, it leads to excessive production of ROS, pro-inflammatory cytokines, and neurotoxic factors, thereby aggravating neuronal harm[95, 101].

In pathological conditions, the phagocytic function of microglia is contingent upon the activation of signaling pathways facilitated by TREM2, a receptor expressed on myeloid cells and osteoblasts [126]. It influences the capacity of microglia to recognize and phagocytose lipids released by dying neurons. Knockout of the TREM2 gene hinders microglia's ability to perceive substances released by dying neurons in the environment, resulting in a decrease in phagocytic function [127]. Ulland et al. demonstrated that the TREM2 knockdown in AD model mice led to dysfunctional and defective energy metabolism in microglia [128]. This dysfunction included decreased expression of glucose transport proteins and glycolytic enzymes, hindered activation of the mTOR-HIF-1α pathway, and impaired microglial adaptability and responsiveness to Aβ accumulation [128]. This finding highlights the correlation between microglia's phagocytic function and their activation state in a neurodegenerative environment. Altered glucose metabolism resulting from impaired microglia activation may derive from their compromised recognition of activation signals or reduced resistance to cytotoxic signals, which can be distinguished by the strength of mTOR signaling [128].

### Hypoxia-induced mitochondrial dysfunction alters microglial glucose metabolism

Hypoxia is a common characteristic of ND, and mitochondria are crucial in oxygen and ROS regulation. Mitochondrial respiration is involved in glucose metabolism in ND, specifically through impaired microglial glycolysis, OXPHOS processes, and insufficient intracellular energy supply. Hypoxia triggers the activation of microglia, affecting their metabolism, ATP production, and mitochondrial function [129, 130]. When subjected to hypoxia, primary microglia in rats undergo activation towards a pro-inflammatory phenotype, causing them to produce pro-inflammatory cytokines and ROS [131-133]. Additionally, hypoxia stimulates the activation of HIF. HIF-1α, a critical component of the hypoxia response, regulates key glycolytic enzymes, such as GLUT-1, HK2 and LDH, contributing to the metabolic shift from OXPHOS to glycolysis in microglia [82, 84]. This shift favors glycolysis as the primary pathway for energy production. The misfolded protein accumulation, like Aβ, α-synuclein, and SOD1, in ND can further elevate ROS levels, triggering oxidative stress [134]. This oxidative stress, in turn, leads to mitochondrial dysfunction and energy depletion, contributing to the disease's progression.

Overall, microglial glycometabolic reprogramming regulates immune activity and brain homeostasis. Changes in the brain microenvironment, cellular metabolic signaling pathways, and mitochondrial malfunction can affect microglia's inflammatory response and phagocytosis, affecting glucose metabolism pathways and ND development. However, the primary and secondary relationships between microglia functional phenotypic changes, changes in glucose metabolic pathways, and the critical timing and molecular targets of these changes are unclear. More in-depth studies could guide the prevention of the onset and progression of ND.

## Therapeutic implications

Given the importance of microglial glucose metabolism in neuroinflammation, regulating microglial glucose metabolism has emerged as a possible therapeutic strategy. Manipulating microglial glucose metabolism through small molecule compounds or genetic strategies can restore cellular homeostasis and mitigate neuroinflammation, ultimately reducing nerve damage and neuronal injury. This section delves into relevant studies concerning treating ND by modulating microglial glucose metabolism (Table 2).

**Table 2:** Therapeutic targets of microglial glucose metabolism in ND.

Intervention	Disease	Target	Effects	Ref.
**Tamoxifen**	AD	PKM2	Inhibits glycolysis	[62]
**INF-γ**	AD	mTOR-HIF-1α	Reversed the defective glycolytic metabolism and inflammatory functions of microglia	[82]
**PGE2**	Aging	EP2-AKT	Promotes the sequestration of glucose into glycogen, reduces glucose flux and mitochondrial respiration	[170]
**TRPV1 agonist**	AD	AKT-mTOR-HIF-1α	Enhances OXPHOS, Inhibits glycolysis	[142, 146]
**2-DG and 3-BPA**	PD	AMPK-mTOR, GLUT1, HK2	Inhibits glycolysis	[46]
**Sodium rutin (NaR)**	AD	Mitochondrial function	Promotes metabolic from anaerobic glycolysis to OXPHOS	[163]
**Running exercise**	AD	GLUT5,TREM2	Inhibits glucose uptake	[152]
**Activate MT 1**	PD	HK2, PDHα1	Positively regulate PDHα1, enhances OXPHOS, Inhibits glycolysis	[66]
**TREM2**	AD	AMPK-mTOR	Inhibits glycolysis	[148]
**G6PD siRNAs**	PD	G6PD-NADPH-ROS	Regulates the pentose phosphate pathway	[95]
**TRPV1 agonist capsaicin**	PD	AKT-mTOR-HIF-1α	Enhances OXPHOS, Inhibits glycolysis	[44]
**Pyrroloquinoline quinone**	PD	LDH	Decrease LDH and NO release	[171]
**TSPO**	AD	HK2	Increases mitochondrial recruitment of HK, induces glycolysis	[159]
**Anti-TLR2 antibody**	AD	PFKFB3	Inhibits glycolysis	[16]
**Retention of iron**	AD	PFKFB3	Promotes glycolysis	[54]
**Aβ1-42**	AD	HK2	Shift from OXPHOS to glycolysis	[166]
**Honokiol**	AD	Glycolysis pathway	Shift from OXPHOS to glycolysis	[172]
**Low-intensity light**	AD	G6PD	Regulates the pentose phosphate pathway	[154]
**α-mangostin**	PD	AMPK-mTOR	Shift glycolysis to OXPHOS	[173]
**Engeletin**	AD	LDH	Reduces LDH and ROS	[157]
**FIR**	AD	ATP	Promotes OXPHOS	[174]
**Hydrogen-rich saline**	ALS	Mitochondrial function	Reduces mitochondrial ROS and enhances mitochondrial ATP	[164]
**Intranasal insulin**	PD	Mitochondrial function	Modulates mitochondrial membrane potential and ROS	[165]
**Clock (Clk)1**	PD	mTOR-HIF-1α and ROS-HIF-1α	Induces aerobic glycolysis	[110]
**ISOA**	AD	NADPH	Suppress NADP+ and NADPH contents, Inhibits ROS accumulation	[175]

AD: Alzheimer’s disease; PD: Parkinson’s disease; ALS: Amyotrophic lateral sclerosis; PKM2: Pyruvate kinase M2; 2-DG: 2-deoxoy-D-glucose; 3-BPA: 3-bromopyruvic acid; PGE2: Prostaglandin E2; TREM2: Triggering receptor expressed on myeloid cells 2; TSPO: Translocator protein; TLR2: Toll-like receptor 2; Thy: Thymidine; 2'-De: 2'-deoxyuridine; FIR: Far Infrared; ISOA: Isoamericanin A; OXPHOS: Oxidative phosphorylation; ATP: Adenosine triphosphate; HK2: Hexokinase 2; PDHα1: Pyruvate dehydrogenase α1; GLUT: Glucose transporters; LDH: Lactate dehydrogenase; ROS: Reactive oxygen species; PFKFB3: 6-phosphofructo-2-kinase/fructose-2,6-bisphosphatase isoform 3; Aβ: β-amyloid; MT1: Melatonin receptor 1; mTOR: Mammalian target of rapamycin; HIF1-α: Hypoxia inducible factor 1- alpha; AKT: Serine-threonine kinase; AMPK: Serine/threonine kinase AMP-activated protein kinase; G6PD: Glucose-6-phosphate dehydrogenase; TRPV1: Transient receptor potential vanilloid type 1; NADPH: Nicotinamide adenine dinucleotide phosphate.

### mTOR-HIF-1α pathway Targeted Therapeutics

As previously described, the AKT-mTOR-HIF-1α and AMPK-mTOR-HIF-1α signaling pathways connect microglial cell glucose metabolism with the inflammatory response. Targeting the mTOR-HIF-1α signaling pathway directly mitigates neuroinflammatory responses in ND. Metformin and rapamycin, two classical inhibitors of mTOR targets, are the main compounds used for this purpose. Both drugs have been discovered to decrease glycolytic, increase OXPHOS in cells, and decrease the production of pro-inflammatory cytokines from microglia [135-137]. However, their mechanisms of action differ. Metformin exerts its effects by acting upstream of mTOR and targeting AMPK, while rapamycin directly inhibits mTOR expression by binding to mTORC1 [135-137]. Metformin, an AMPK activator, has demonstrated the ability to improve glucose uptake and alleviate symptoms of AD [138]. By activating TSC2, a negative regulator of mTORC1, and inhibiting the mTORC1 component RAPTOR5, AMPK reduces mTORC1 expression [92, 93]. Elevated AMPK activity during energy shortage leads to decreased mTOR activity.

In an ALS model, treatment with rapamycin reduced microglial proliferation, increased anti-inflammatory factor expression, and slowed disease progression [139]. Despite rapamycin being recognized as a specific inhibitor of mTORC1, prolonged exposure also influences the expression of mTORC2 [140]. mTORC2 plays a role in in activating the AKT pathway, which has the ability to counteract neuronal cell death in experimental models of ND [140]. As a result, extended rapamycin treatment may impact mTORC2 expression, leading to a reduction in AKT activation and compromising neuronal survival. It is difficult to inhibit mTORC1 specifically to treat neurodegeneration, necessitating a careful study of rapamycin's dose and administration schedule [141].

Besides metformin and rapamycin, several other drugs have demonstrated the ability to modulate the mTOR-HIF-1α pathway in microglia as a potential treatment for ND. Capsaicin, for example, interacts with the transient receptor potential vanilloid 1 (TRPV1) channel and exhibits neuroprotective properties in AD and PD patients [142-144].

Research studies have indicated that capsaicin, functioning as a TRPV1 agonist, can prevent the degeneration of DA neurons by inhibiting oxidative stress and neuroinflammation induced by the activation of glial cells in PD models [145]. This protective mechanism may entail capsaicin's pharmacological stimulation of TRPV1, which targets the mTOR-HIF-1α pathway. Capsaicin restores microglial energy metabolism, improves phagocytosis, and facilitates the degenerative processes identified in AD and PD disease models [44, 142, 146, 147].

The microglial regulator TREM2 is another key mTOR target, capable of regulating mTOR activity [128]. TREM2 failure causes anomalies in the downstream mTOR signaling pathway, resulting in defective microglial glycolysis, poor phagocytosis, and increased Aβ plaque formation. Thus, drugs that interfere with TREM2 may effectively improve the symptoms of AD [128, 148-150]. The emerging recognition of the substantial involvement of TREM2 in ND has prompted the hypothesis that it holds promise as a potential therapeutic approach [128, 150, 151]. A non-pharmacological intervention involving running exercise has been discovered, which effectively suppresses the shedding of TREM2[152]. This exercise-induced inhibition subsequently enhances microglial glucose metabolism and morphological plasticity within the hippocampus of mice with AD [152]. Consequently, this novel therapeutic strategy may offer a viable avenue for the treatment of AD. However, the comprehension of TREM2's functionality remains limited, and its precise association with neurological disorders has not been fully elucidated. Currently, there are no *in vivo* examples of drugs specifically targeting TREM2 agonism for treating ND like AD and PD, and this approach may still present challenges [151].

Furthermore, IFN-γ has been reported as a potential treatment for AD models. *In vivo* and *in vitro* injection of IFN-γ targets mTOR, rescuing microglia from defective glycolytic metabolism and restoring their anti-inflammatory function. This, in turn, increases the phagocytosis of Aβ and improves cognitive deficits [82, 153]. 2-deoxy-D-glucose (2-DG) and 3-bromopyruvic acid (3-BPA) are two small molecule drugs commonly used to study the regulatory mechanisms of glycolytic pathways. Both drugs effectively inhibit mTOR phosphorylation in microglia, reducing glycolysis and ameliorating MPTP-induced neuroinflammation in PD model mice [46]. Due to the mTOR pathway's broad prevalence in numerous cell types, these drugs only partially inhibit it and may affect other cells and biological processes. Thus, specifically targeting the microglia mTOR signaling pathway for treating ND continues to pose challenges.

### Glycolytic enzyme-targeted therapeutics

As previously discussed, microglial glycolysis is regulated by multiple key enzymes crucial in neuroinflammation expression. The targeting of these enzymes presents a potentially effective strategy for the treatment of ND. Notably, G6PD, an enzyme abundantly expressed in microglia, has been implicated in the degeneration of DA neurons induced by neuroinflammation [95]. Therefore, targeting G6PD shows promise as a potential treatment for ND. In this regard, photo biomodulation, a noninvasive approach, is being considered as a viable method for G6PD therapy. The application of low-intensity light has been found to reduce Aβ-induced microglial G6PD activity, NOX expression, and modulate the rate of the PPP, consequently inhibiting the production of ROS and preventing neuronal death [154]. NOX is known to activate microglia and contribute to dopaminergic neurodegeneration in mouse models of PD. This detrimental process can be counteracted by the natural compound apocynin, which effectively inhibits oxidative stress and neuroinflammation, thereby exerting neuroprotective effects [155].

Several *in vitro* experiments have also demonstrated the potential of pharmacological interventions targeting key enzymes of glycolysis, including PFK, HK2, and LDH, to modulate microglial glycolysis and alleviate neuroinflammatory responses (Table 2). For instance, in a traditional Chinese medicine study, essential oil suppressed glycolysis in BV2 microglia by inhibiting PFK and pyruvate kinase (PK) activities [156]. This inhibition decreased the pro-inflammatory factors secretion, like IL-6, IL-1β, and TNF-α [156]. Similarly, Engeletin demonstrated the ability to inhibit Aβ1-42-induced toxicity and LDH release in BV-2 microglial cells [157]. It attenuated oxidative stress and suppressed the production of inflammatory factors like IL-1β, TNF-α, and iNOS [157]. However, most of these chemicals and medications are still in early lab *in vitro* research. Therefore, further studies and evaluations are necessary to determine their potential and efficacy in treating neuroinflammation.

### Mitochondria function targeted therapeutics

The role of mitochondrial function in the metabolic activity and inflammatory regulation of microglia is of utmost importance, thus warranting significant attention in the development of potential treatments for ND. The high expression of the translocator protein (TSPO) in the outer membrane of mitochondria is widely recognized as a characteristic feature of neuroinflammation, particularly in activated microglia [158]. In an AD model, the loss of TSPO function hampers mitochondrial respiration, resulting in an augmented mobilization of mitochondria to the HK enzyme. Consequently, this alteration in microglial metabolism towards glycolysis leads to a reduction in phagocytic activity [159]. However, it's worth noting that the experimental approach employed in this study involved the knockdown of TSPO.

While certain TSPO ligand compounds have demonstrated anti-inflammatory and neuroprotective effects through inhibiting TSPO expression and activity in microglia within AD models [160-162], the precise mechanism of action and the impact on microglial mitochondrial function necessitate additional elucidation. Therefore, further evaluation of TSPO's potential and efficacy in regulating microglial function is necessary.

Several drugs targeting microglial mitochondrial function have shown promise in *in vivo* models of neurodegenerative diseases. Sodium rutin (NaR), a natural flavonoid, enhances microglial mitochondrial OXPHOS, providing enough ATP for Aβ clearance [163]. NaR also ameliorates synaptic plasticity damage and exhibits neuroprotective effects in both in vitro and *in vivo* models of AD [163]. Hydrogen-rich saline, an antioxidant, protects mitochondrial function by reducing ROS formation and enhancing mitochondrial ATP synthesis[164]. In mutant SOD1 G93A mice, it significantly inhibits microglia activation, delaying the onset of ALS disease and prolonging survival [164]. Furthermore, insulin, a commonly used drug, when administered through the intranasal pathway, improves mitochondrial functional indices, modifies mitochondrial biogenesis and fission, and activates microglia in a 6-hydroxydopamine (6-OHDA)-induced PD rat model [165]. This intervention results in an alleviation of motor dysfunction and a reduction in dopaminergic cell death.

In summary, while some drugs and gene modulation strategies have shown potential therapeutic effects interfering with microglial glucose metabolism pathways, they are still in the laboratory research and evaluation phase. Their efficacy and feasibility in treating ND require more investigation and clinical trials. These therapies' effective doses and treatment regimens need further research. Achieving specific targeting of microglia remains a challenging issue currently. Therefore, it is essential to construct a screening platform for key enzymes of glucose metabolism and related signaling pathways and develop more tissue-specific targeted small molecule drugs.

## Conclusion

The central inflammation observed in ND is largely attributed to the actions of microglia. These microglia cells possess the ability to adopt various phenotypes in response to different stimuli, and during the pathological progression of these diseases, notable alterations in glucose metabolism can be observed. By manipulating the glycometabolic reprogramming of microglia, it becomes feasible to modulate their functionality and potentially reverse the central inflammatory response associated with ND. Glycometabolic reprogramming, as an emerging field, provides an important contribution to our understanding of microglia function and immune responses. Targeting microglia metabolism may be a valuable direction for developing therapies for ND. However, there remains a significant knowledge gap regarding the precise implications of microglial glucose metabolism in the context of ND. Further investigation is required to comprehensively explore the specific mechanisms underlying this phenomenon. Additionally, the presence of immune tolerance and the dynamic alterations in microglia phenotype during pathological processes pose challenges in the development of targeted drugs aimed at regulating glucose metabolism. In summary, microglia glycometabolic reprogramming regulates the prevention and treatment of ND, although more mechanistic research is needed.
